# Targeting metabolic flexibility by simultaneously inhibiting respiratory complex I and lactate generation retards melanoma progression

**DOI:** 10.18632/oncotarget.6134

**Published:** 2015-10-15

**Authors:** Balkrishna Chaube, Parmanand Malvi, Shivendra Vikram Singh, Naoshad Mohammad, Avtar Singh Meena, Manoj Kumar Bhat

**Affiliations:** ^1^ National Centre for Cell Science, Savitribai Phule Pune University Campus, Ganeshkhind, Pune, India; ^2^ Current address: Department of Physiology, University of Tennessee Health Science Center, Memphis, USA

**Keywords:** melanoma, complex I, LDH, metabolic catastrophe, synthetic lethality

## Abstract

Melanoma is a largely incurable skin malignancy owing to the underlying molecular and metabolic heterogeneity confounded by the development of resistance. Cancer cells have metabolic flexibility in choosing either oxidative phosphorylation (OXPHOS) or glycolysis for ATP generation depending upon the nutrient availability in tumor microenvironment. In this study, we investigated the involvement of respiratory complex I and lactate dehydrogenase (LDH) in melanoma progression. We show that inhibition of complex I by metformin promotes melanoma growth in mice via elevating lactate and VEGF levels. In contrast, it leads to the growth arrest *in vitro* because of enhanced extracellular acidification as a result of increased glycolysis. Inhibition of LDH or lactate generation causes decrease in glycolysis with concomitant growth arrest both *in vitro* and *in vivo*. Blocking lactate generation in metformin-treated melanoma cells results in diminished cell proliferation and tumor progression in mice. Interestingly, inhibition of either LDH or complex I alone does not induce apoptosis, whereas inhibiting both together causes depletion in cellular ATP pool resulting in metabolic catastrophe induced apoptosis. Overall, our study suggests that LDH and complex I play distinct roles in regulating glycolysis and cell proliferation. Inhibition of these two augments synthetic lethality in melanoma.

## INTRODUCTION

Malignant melanoma is one of the most aggressive forms of skin cancer with high metastatic potential and resistance to many cytotoxic agents [1, 2]. Despite extensive research and partial successes gained by the use of currently available drugs there is no effective treatment against malignant melanoma [1-3]. Melanoma cases are increasing every year and account for about 75% of skin cancer-related deaths worldwide [2, 3]. Poor response to currently available therapeutic options and development of resistance to therapy warrant exploration of new strategies to treat melanoma.

Enhanced aerobic glycolysis is a characteristic feature of many cancers [3-5]. It has been reported that melanoma cells, owing to the BRAF mutation, depend majorly on glycolysis for ATP generation, and exhibit dysfunctional oxidative phosphorylation [6, 7]. Cancer cells derive ATP, biosynthetic intermediates, and reducing equivalents by unusually engaging in biochemical pathways such as glycolysis, glutaminolysis, and the pentose phosphate pathway [5]. Normal (non-cancerous) cells derive ATP primarily through mitochondrial OXPHOS; while cancer cells rely mainly on aerobic glycolysis to generate ATP and glycolytic intermediates those facilitate rapid growth [4, 5]. Enhanced lactate generation has been correlated with aggressiveness of cancer. Numerous studies have identified lactate dehydrogenase (LDH), which catalyzes the conversion of pyruvate to lactate, as the most consistent marker of the aggressive and rapidly growing cancers [8-11]. LDH plays an important role in regulating glycolysis, maintaining cellular redox state, mitochondrial physiology and tumor maintenance [12]. Altered metabolism of cancer cell can be associated with mitochondrial dysfunction which involves inhibition of OXPHOS, increase in reactive oxygen species (ROS) and promoting uncontrolled growth, that in turn further supports the development of a metastatic phenotype [13,14]. Respiratory complex I is the largest and most complex enzyme which catalyzes oxidation of NADH in electron transport chain [15]. Complex I plays important role in ATP generation in normal cells and is a main site of ROS generation, however, its role in tumorigenesis is largely unclear. Most of the reports suggest that complex I activity is suppressed in cancer cells and its inhibition promotes proliferation and metastasis [16-18]. Conversely, it has also been reported that melanoma cells display increased OXPHOS function which causes drug resistance [19-21]. Owing to the metabolic flexibility in choosing various metabolic pathways, particularly glutamine metabolism [22], melanoma cells can escape from perturbation of a molecule in a metabolic pathway [23]. Therefore, more than one molecules of different metabolic pathways need to be targeted for an effective and curative outcome.

Biguanides like metformin and phenformin are known to inhibit respiratory complex I. Metformin, a first line therapy for patients with type 2 diabetes mellitus (T2DM), is a member of the biguanides and has been recently discovered to have anti-tumorigenic activity [24, 25]. At physiological level, metformin exerts its biological activity by decreasing hepatic glucogenesis, increasing insulin sensitivity, elevating peripheral glucose uptake and reducing absorption of glucose from the gastrointestinal tract [26]. At the cellular level, metformin works primarily by inhibiting mitochondrial complex I, which impedes oxidative phosphorylation resulting in decreased ATP level leading to activation of AMPK [27-30]. Moreover, it has been shown that metformin decreases mitochondrial respiration coupled with ATP generation, thereby causing an increase in glycolysis [31, 32]. The anticancer action of metformin has been shown in various cancers with distinct mechanisms [33, 34]. However, role of metformin in melanoma is not much clear. Unlike other cancer types, metformin is shown to have both tumor promoting as well as antitumor activity in melanoma [35-37]. Also, metformin, in combination with BRAF inhibitors, has been shown to suppress melanoma growth [38].

In the present study, with the speculation that disrupting ATP generating routes by inhibiting complex I and LDH together could be synthetically lethal to melanoma cells, we used metformin or phenformin and oxamate/dichloro acetate (DCA) to inhibit these two enzymes using both cell and animal models. We demonstrate that inhibition of complex I and LDH activity have distinct impact on cell growth and proliferation. Interestingly, inhibition of complex I by metformin further promotes aerobic glycolysis resulting in enhanced tumor growth in mice. Ablation of metformin-induced lactate generation by using oxamate or DCA leads to cytostasis and/or apoptosis in melanoma cells.

## RESULTS

### Metformin exhibits distinct *in vitro* and *in vivo* actions on growth of melanoma

**Figure 1:**
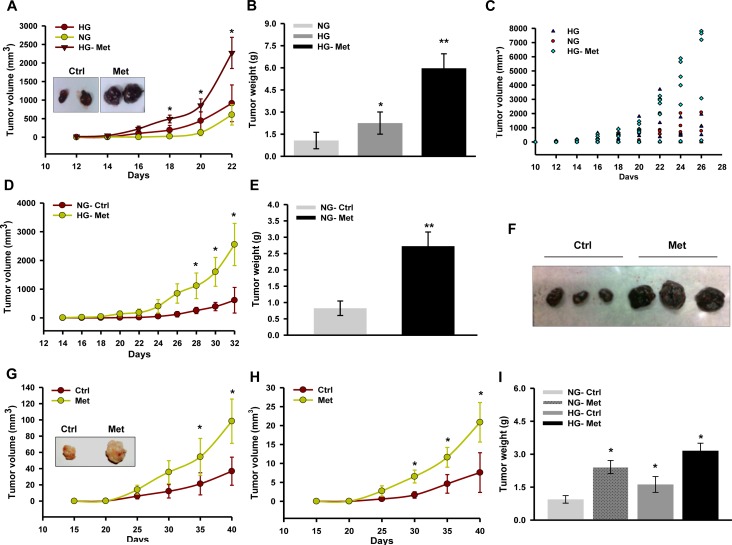
Metformin promotes melanoma tumor growth in mice **A.**-**C.** C57BL/6J mice were made diabetic by injecting 50 mg/kg streptozotocin (STZ) intraperitoneally for 3 consecutive days. Tumor was developed by injecting 1 × 10^5^ B16F10 melanoma cells subcutaneously in C57BL/6J mice, one week post STZ injection. Metformin (200 mg/kg) was administered in hyperglycemic mice orally before injecting the cells. Graph representing tumor progression **A.**, tumor weight **B.**, and tumor volume of individual mice **C.** in hyperglycemic mice. Results are given as means ± SD (*n* = 7). **D.** Tumor progression of B16F10 isograft in normoglycemic mice. Mice were administered with metformin (200 mg/kg) orally after the appearance of palpable tumor. **E.** and **F.** Weight and representative image of tumors excised from normoglycemic mice administered with or without metformin. Results are given as means ± SD (*n* = 5). **G.** Tumor progression of A375 xenograft in hyperglycemic mice administered with or without metformin (200 mg/kg). Results are given as means ± SD (*n* = 5). **H.** Tumor progression of A375 xenograft in normoglycemic mice administered with or without metformin (200 mg/kg). Results are given as means ± SD (*n* = 5). **I.** Weight of tumors excised from either hyperglycemic or normoglycemic mice administered with or without metformin. The values *p < 0.05, **p < 0.01 denote significant differences between the groups. (HG- hyperglycemic, NG- normoglycemic, Ctrl- control, Met- metformin)

Metformin suppresses tumor growth by inhibiting complex I which is influenced by glucose [30]. Moreover, glucose is known to alter the activity of respiratory enzymes [39]. Therefore, to explore the consequence(s) of complex I inhibition and influence of glucose on action of metformin on melanoma progression, we monitored isograft/xenograft progression in streptozotocin (STZ) induced hyperglycemic mice. We noted that metformin promoted B16F10 derived isograft progression in hyperglycemic mice as compared to untreated control (Figure 1A, 1B and 1C). Also, metformin positively influenced progression of tumor in normoglycemic C57BL/6J mice (Figure 1D, 1E and 1F). Similarly, oral administration of metformin promoted growth of A375 xenograft in hyperglycemic as well as in normoglycemic NOD/SCID mice as compared to untreated control (Figure 1G, 1H and 1I).

**Figure 2:**
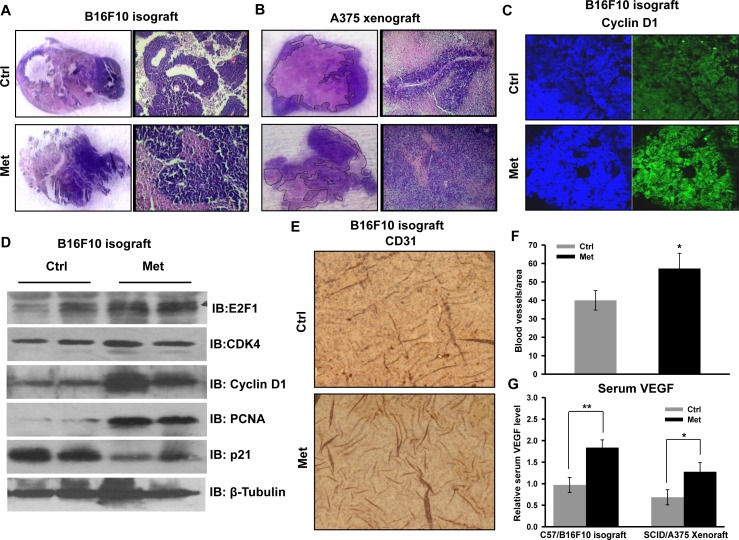
Metformin promotes melanoma tumor growth by inducing angiogenesis and by inhibiting necrosis **A.** and **B.** Representative H&E images of tumor sections of B16F10 isograft **A.** and A375 xenograft **B.** from control and metformin groups showing necrotic regions (pink) and healthy cells (blue). **A.** H&E staining of B16F10 isograft section from control and metformin group. **B.** H&E staining of A375 xenograft section from control and metformin groups. **C.** Representative immunoblots showing the expression of indicated cell cycle regulatory molecules in the lysate of B16F10 derived tumors from control and metformin groups. **D.** Representative immunohistochemical image showing the expression of cell cycle regulatory molecule cyclin D1 in the B16F10 isograft section from control and metformin groups. **E.** and **F**. Immunohistochemical analysis of angiogenesis marker CD31 in the tumor section of B16F10 isograft of control and metformin group **G.** Relative serum level of VEGF in control and metformin administered C57 and NOD/SCID mice. All these experiments were performed in normoglycemic mice. Data were represented as means ± SD. The values *p < 0.05, **p < 0.01 denote significant differences between the groups. (HG- hyperglycemic, NG- normoglycemic, Ctrl- control, Met- metformin).

To check the cellular and molecular events associated with increased tumor progression, tumor sections were examined for histopathological analysis. High cell density and reduced necrosis were clearly visible in the sections of both tumor types (B16F10 derived isograft as well as A375 derived xenograft) from metformin administered mice (Figure 2A and 2B). We noted that metformin enhanced proliferation and progression of A375 derived xenograft was phenotypically distinct as compared to the control tumor. This is suggestive of a grade advancement of primary tumor, evident by elongated morphology of nuclei compared to rounded morphology in the control tumors sections (unpublished information). Immunohistochemical staining of the cell cycle regulatory protein cyclin D1 (Figure 2C) was found to be higher in tumor section from metformin administered mice. Further, we validated the enhanced tumor growth by checking status of cell cycle regulatory proteins by immunoblotting of tumor lysates. We found that levels of molecules cyclin D1, CDK4, E2F1 and PCNA were increased significantly in the tumor lysates of metformin administered mice as compared to control, while p21 level was diminished (Figure 2D). These results indicate that metformin, irrespective of glycemic status of mice, promotes melanoma growth by modulating cell cycle regulatory proteins. Moreover, immunohistochemical analysis of tumor sections strengthened this observation, because metformin treatment enhanced protein levels of CD31, an endothelial marker (Figure 2E and 2F), and increased the serum level of VEGF (Figure 2G), suggesting that metformin promotes angiogenesis in melanoma tumors.

Next, we checked effect of metformin on the growth and proliferation of melanoma cells *in vitro*. In contrast to our *in vivo* findings, metformin treatment resulted in growth suppression of melanoma cells *in vitro* (Supplementary Figure S1A, S1B, S1C and S1D). Thereafter, we investigated the impact of complex I inhibition using metformin and phenformin on melanoma cells growth as both of these inhibited complex I activity (Supplementary Figure S2). We found that metformin and phenformin caused growth arrest in melanoma cells grown under high glucose. However, in presence of low glucose, treatment with these agents resulted in cell death (Supplementary Figure S3A, S3B, S3C and S3D). It is likely that metformin mediated growth arrest is due to reduction in glucose level and extracellular acidification, of media (Supplementary Figure S4A and S4B). Interestingly, replacing the medium after every 12 h with fresh medium containing 25 mM glucose increased clonogenic survival upon metformin treatment. As metformin-treated cells utilized glucose very rapidly in comparison to the control; therefore, replenishment of medium is essentially required to maintain glucose level and pH thereby survival of cells in the presence of metformin (Supplementary Figure S4C and S4D). These results suggest that concentration of glucose available in the culture medium influences metformin's action.

### Inhibition of respiratory complex I activity promotes aerobic glycolysis

**Figure 3:**
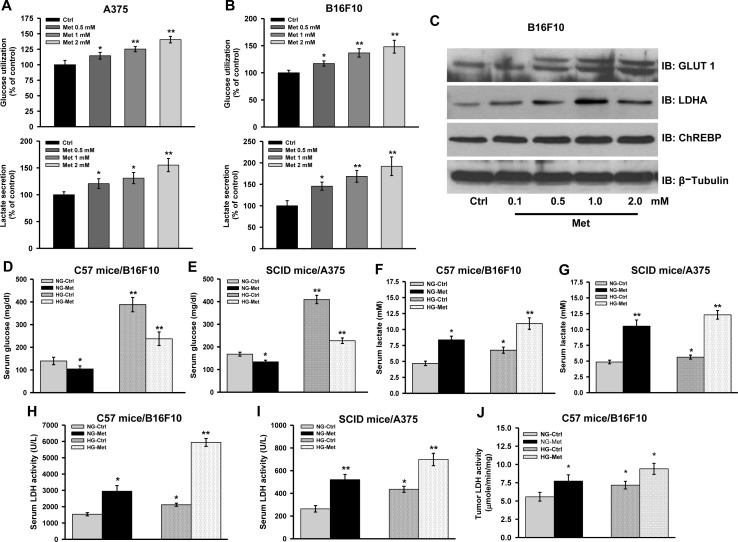
Complex I inhibition induces aerobic glycolysis **A.** and **B.** A375 and B16F10 cells were cultured in presence of different concentrations of metformin (0.5, 1.0, and 2.0 mM) for 36 h. Glucose and lactate level in the spent culture medium was determined by using enzymatic assay kits. **C.** Representative immunoblots showing protein level of indicated molecules in whole cell lysate of B16F10 cells treated with metformin for 36 h. **D.** and **E.** Relative level of glucose in serum collected from normoglycemic or hyperglycemic C57BL/6J mice bearing B16F10 isograft **D.** and NOD/SCID mice bearing A375 xenograft **E.** from metformin administered groups (*n* = 4). **F.** and **G.** Relative levels of lactate in serum collected from normoglycemic or hyperglycemic C57BL/6J mice bearing B16F10 isograft **F.**, and NOD/SCID mice bearing A375 xenograft **G.** from metformin administered groups (*n* = 4). **H.** and **I.** Serum LDH activity in normoglycemic or hyperglycemic C57BL/6J mice bearing B16F10 isograft **H.** and NOD/SCID mice bearing A375 xenograft **I.** from control and metformin administered groups (*n* = 4). **J.** LDH activity in the B16F10 isograft from metformin administered normoglycemic and hyperglycemic mice. Values are represented as mean ± SD. The values *p < 0.05, **p < 0.01 denote significant differences between the groups (*n* > 3 at the least). (NG-normoglycemic, HG-hyperglycemic, Ctrl- control, Met-metformin).

Discrepancy in the outcome of metformin treatment *in vivo* and *in vitro* prompted us to explore the cause for the same. It is known that metformin exerts its action primarily by inhibiting mitochondrial OXPHOS enzyme complex I [27-29] and inhibition of complex I results in increased tumor growth and proliferation [14-18]. As we observed that metformin treatment caused acidification of culture medium due to enhanced aerobic glycolysis (Supplementary Figure 4A and 4B), we therefore speculated that this might be the reason for cell growth arrest. Thus, we measured level of glucose as well as lactate in the spent culture medium collected from control and metformin treated cells. Increased glucose utilization (as evident by the presence of less residual glucose in medium) and higher lactate level was detected in metformin treated cells as compared to the control (Figure 3A and 3B).

To strengthen these findings, we further explored the expression pattern and activity of some of the proteins and enzymes involved in regulating glycolysis. We found that metformin increased protein levels of key glycolytic molecules GLUT1, LDHA and ChREBP in a concentration dependent manner (Figure 3C). These results suggest that inhibition of complex I activity by metformin could possibly enforce cancer cells to adopt glycolytic route for ATP generation causing enhancement in tumor progression in mice.

To evaluate whether glycolytic phenotype can also be induced *in vivo* upon inhibition of complex I, we first checked the level of glucose in serum collected from mouse bearing tumor along with normoglycemic mice treated or untreated with metformin. Significant decline in the level of serum glucose was observed in metformin administered normoglycemic, tumor bearing mice (104.3 ± 13.7 mg/dl in C57BL/6J, and 134.0 ± 7.2 mg/dl in NOD/SCID) as compared to untreated control (139.7 ± 16.3 mg/dl in C57BL/6J, and 168.0 ± 8.5 mg/dl in NOD/SCID) (Figure 3D and 3E). Similarly, metformin treatment caused reduction in serum glucose in tumor bearing hyperglycemic mice (Met, 237.3 ± 30.0 mg/dl in C57BL/6J, and 227.3 ± 12.3 mg/dl in NOD/SCID) as compared to control (388 ± 31.5 mg/dl in C57BL/6J, and 409.3 ± 18.6 mg/dl in NOD/SCID mice) (Figure 3D and 3E). Secretion of lactate is a hallmark of glycolytic tumors. Therefore, we checked lactate level which was found to be very high in the serum of tumor bearing normoglycemic as well as hyperglycemic mice administered with metformin (Figure 3F and 3G). Elevated serum LDH is associated with high grade aggressive tumors and poor prognosis in case of metastatic melanoma [8-10]. Thus, serum LDH activity was monitored and was found to be very high in the serum collected from hyperglycemic and normoglycemic mice administered with metformin as compared to control (Figure 3H and 3I). Furthermore, increased LDH activity was detected in tumor tissues of metformin administered mice compared to the untreated control (Figure 3J).

### Inhibition of complex I together with LDH or lactate generation is synthetically lethal to melanoma cells

**Figure 4:**
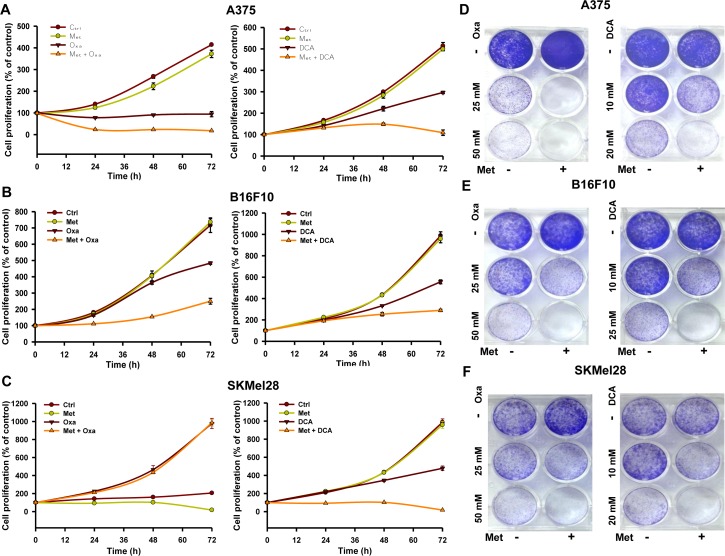
Inhibition of complex I sensitizes cancer cells to LDH and PDK1 inhibitors oxamate and DCA **A.**-**C.** Effect of oxamate and DCA on growth of A375, B16F10 and SKMel28 cells treated with or without metformin. Cells were treated with 25 mM oxamate or 10 mM DCA either alone or with 2 mM metformin for indicated time point. Cell viability was assessed by MTT assay. **D.**-**F.** Survival of A375 **D.**, B16F10 **E.** and SKMel28 **F.** cells grown in the presence of oxamate (25 or 50 mM) or DCA (10 or 20 mM) alone or together with 2 mM metformin for 48 h. Representative images showing the long term survival of cancer cells treated with indicated concentrations of DCA and oxamate alone or together with metformin (2 mM) and allowed to grow for 48 h. Medium was replaced with fresh medium without any inhibitors. Medium was changed every 2-3 days and allowed to grow in drug free medium for further 10-15 days. Colonies were stained with crystal violet and photographed. All values are represented as mean ± SD. (Ctrl- control, Met- metformin, Oxa- oxamate).

To explore the possibility whether metformin promoted glycolysis or excess lactate generation is the major cause of increased growth and proliferation of melanoma tumors, lactate generation was inhibited by using LDH inhibitor oxamate and PDK1 inhibitor DCA. We found that oxamate and DCA alone have inhibitory effect on melanoma cell growth (Figure 4A, 4B and 4C; Supplementary Figure S5). Reduced cell survival was observed upon treatment with oxamate or DCA together with metformin (Figure 4A, 4B and 4C). Similarly, other well-known glycolytic inhibitors like phloretin (GLUT1 inhibitor), 2-deoxy-D-glucose (2DG), and iodoacetate also restricted proliferation of melanoma cells treated with metformin (Supplementary Figure S6A and S6B). Enhanced glucose uptake through GLUT1 and increased aerobic glycolysis through LDHA and PDK1 is the hallmark of cancer cells. Therefore, we chose oxamate and DCA over other glycolytic inhibitors.

**Figure 5:**
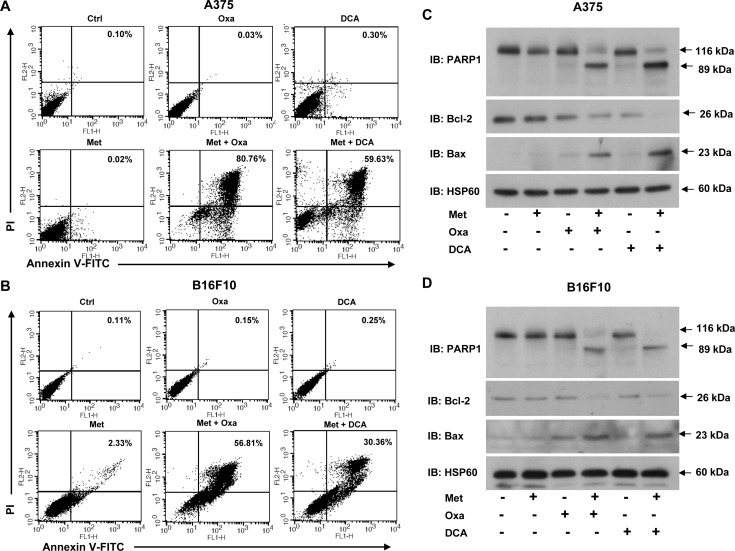
Simultaneous blocking of lactate generation and complex I induces apoptosis in melanoma cells **A.** and **B.** A375 and B16F10 cells were grown in DMEM with or without 50 mM oxamate and 20 mM DCA either alone or together with 2 mM metformin for 36 h. Apoptosis was detected by using Annexin V and PI dual positive cells via flow cytometry. **C.** and **D.** Representative immunoblots showing the protein levels of apoptotic markers PARP1, Bax and antiapoptotic molecule Bcl-2 in A375 **C.** and B16F10 cells **D.** grown under above mentioned conditions. HSP60 was used as a loading control. (Ctrl- control, Met- metformin, Oxa- oxamate).

Next, to check the long term survivability of melanoma cells in the presence of metformin and oxamate/DCA alone or in combination together, we performed clonogenic survival assay. No surviving colonies were detected in melanoma cells treated with oxamate and metformin together (Figure 4D, 4E and 4F). Similar results were also obtained in cells treated with metformin and DCA together (Figure 4D, 4E and 4F). Subsequently, we checked whether inhibition of these two enzymes induced death in melanoma cells as evident by Annexin V and PI staining. All these agents alone caused growth arrest, but did not induce apoptosis. However, increased apoptosis was detected in A375 and B16F10 cells treated with oxamate/DCA together with metformin as compared to single agent alone (Figure 5A and 5B). This observation was further confirmed by immunoblotting for the apoptotic markers PARP1, Bax and Bcl-2 (Figure 5C and 5D). Increased PARP cleavage and Bax level while decreased level of antiapoptotic protein Bcl-2 was detected in cells treated with metformin and oxamate/DCA together as compared to single agent alone (Figure 5C and 5D). These results indicate that inhibition of complex I and LDH together can promote induction of apoptosis.

Next, we have checked the impact of metformin and oxamate/DCA either alone or in combination on the survival of non-cancerous cells. We used three different non-cancerous cell lines namely AML12 (mouse hepatocytes), L6 (rat muscle cells) and MEF (mouse embryonic fibroblasts) to test the effect of combination treatment. Although, combination of metformin and oxamate/DCA slackened the proliferation of these cells (Supplementary Figure S7A, S7B and S7C), it did not affect the cell viability (Supplementary Figure S7D), indicating that this combination is effective in selectively killing cancer cells.

### Blocking complex I and lactate generation imposes metabolic catastrophe

**Figure 6:**
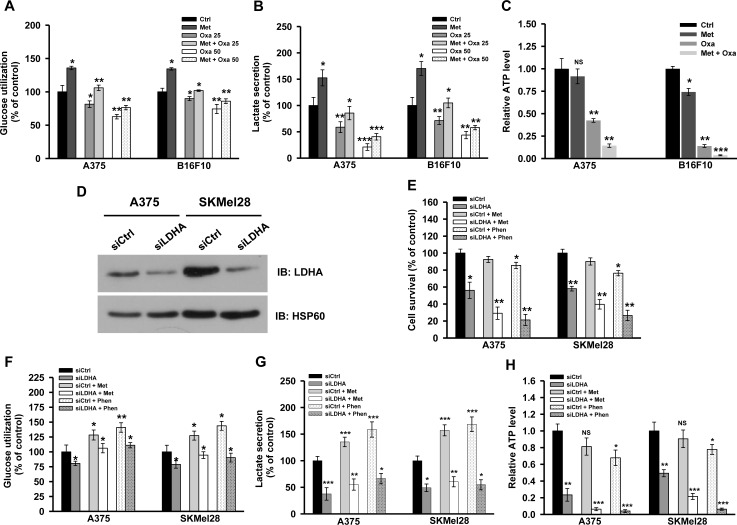
Inhibition of complex I and lactate generation together induces metabolic catastrophe **A.** and **B.** A375 and B16F10 cells were treated with oxamate (25 and 50 mM) either alone or with 2 mM metformin for 36 h. Level of glucose and lactate in the culture medium was measured as mentioned in method section. **C.** Relative ATP levels in A375 and B16F10 cells grown in the conditions mentioned in **A.**. **D.** Human melanoma cells (A375 and SKMel28) were grown in 96 well plates (60% confluency) and transfected with control and LDHA specific siRNA as described in methods section. These cells were treated with either metformin (2 mM) or phenformin (100 μM) for additional 48 h. Inhibition of LDHA expression was confirmed by immunoblotting. **E.** Survival of A375 and SKMel28 upon knocking down LDHA grown in presence or absence of metformin and phenformin. **F.**-**H.** A375 and SKMel28 cells were grown in 35 mm culture dish for 24 h. These cells were transfected with either control or LDHA specific siRNA and allowed to grow for further 24 h. Cells were treated with either 2 mM metformin or 100 μM phenformin for additional 48 h. Glucose utilization **F.**, lactate secretion **G.** and total cellular level of ATP **H.** was determined in melanoma cells as described in method section. Values are represented as mean ± SD. The values *p < 0.05, **p < 0.01, ***p < 0.001 denote significant differences between the groups (*n* > 3 at the least). (Ctrl- control, Met- metformin, Oxa- oxamate, Phen- phenformin).

To ensure whether oxamate and metformin co-treatment affects metabolism of melanoma cells, we measured metabolic parameters in presence of these inhibitors. Metformin treatment elevated glycolysis, while oxamate treatment resulted in decrease in glucose utilization and lactate secretion (Figure 6A and 6B). Interestingly, glucose utilization and lactate secretion was inhibited significantly upon treating cells with both oxamate and metformin together (Figure 6A and 6B). Additionally, both oxamate as well as metformin reduced ATP levels by inhibiting substrate level phosphorylation and OXPHOS respectively (Figure 6C). Also, a significant decline in ATP level was detected in cells treated with oxamate and metformin together (Figure 6C). Further, to confirm the involvement of LDH in melanoma cell growth and proliferation, knock down of LDHA (LDHA-KD) was performed using specific siRNA (Figure 6D). Increased cell death was observed in LDHA-KD cells treated with metformin or phenformin (Figure 6E). A significant reduction in glycolysis (as evident by decreased glucose utilization and lactate secretion) and ATP level was observed in LDHA-KD cells as compared to control (Figure 6F, 6G and 6H). Moreover, decrease in glycolytic rate as well as a sharp decline in ATP level was observed in LDHA-KD cells treated with either metformin or phenformin (Figure 6F, 6G and 6H). These results indicate that simultaneous inhibition of both complex I and LDH or lactate generation pathway induces metabolic catastrophe owing to the disruption of cellular ATP pool, eventually leading to growth arrest and cell death.

### Simultaneous inhibition of complex I and LDH by metformin and oxamate retards tumor progression in mice

**Figure 7:**
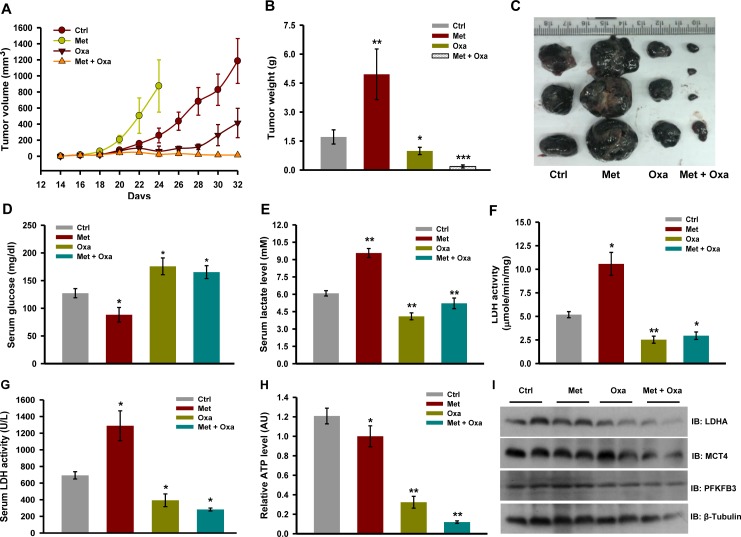
Inhibition of LDH activity restricts tumor progression of melanoma tumor upon complex I inhibition **A.** Progression of B16F10 isograft in C57BL/6J mice administered with metformin (200 mg/kg, orally) and oxamate (500 mg/kg, orally) either alone or together (*n* = 5). Values are represented as mean ± SD. **B.** and **C.** Tumor weight and representative image of tumors excised from mice of indicated groups. **D.** and **E.** level of glucose and lactate in the serum collected from tumor bearing mice with indicated treatment groups (*n* = 4) **F.** Enzymatic activity of LDH in tumor lysate **G.** LDH activity in serum from the mice of indicated treatment groups (*n* = 3). **H.** Relative ATP levels in the tumors of indicated treatment groups (*n* = 3). **I.** Immunoblots showing protein levels of indicated molecules in the whole tumor lysates. Values are represented as mean ± SD. The values *p < 0.05, **p < 0.01 denote significant differences between the groups. (Ctrl- control, Met- metformin, Oxa- oxamate).

To verify whether combination of oxamate and metformin is also effective in diminishing tumor progression, tumors were developed by injecting B16F10 cells in C57BL/6J mice. After tumors reached up to 50 mm^3^ in size, mice were randomized into four groups; (a) control, (b) metformin, (c) oxamate, and (d) metformin and oxamate together treated mice. We noticed that mice administered with metformin developed larger tumor as compared to the untreated control (Figure 7A, 7B and 7C). While in mice administered with oxamate, tumors progressed at a slower rate than the untreated control as well as in mice administered with metformin (Figure 7A). Interestingly, we observed that oxamate retarded tumor progression dramatically when administered together with metformin as evident by reduction in tumor volume and tumor weight (Figure 7A, 7B and 7C).

Further, to confirm whether abrogation of metformin induced tumor progression by oxamate is a consequence of reduction in aerobic glycolysis, we measured metabolic parameters *in vivo*. Metformin administered mice had low serum glucose, higher lactate and LDH levels in serum as compared to control, whereas relatively higher serum glucose and low lactate level were detected in the mice treated with oxamate alone or metformin together with oxamate (Figure 7D and 7E). Activity of LDH enzyme was significantly diminished in the tumors excised from mice treated with oxamate alone or metformin together with oxamate (Figure 7F). Relatively very low level of ATP was detected in the tumors of mice administered with both oxamate and metformin together as compared to single agent alone. However, we did not observe significant changes in the protein levels of molecules regulating glycolysis (Figure 7I). Importantly, no significant change in body weight was detected in the mice receiving combination treatment and no possible adverse side effects or gross symptoms of generalized toxicity were observed upon visual inspection (Supplementary Figure S8A). Moreover, seemingly no pathological abnormalities were observed in the vital organs like lung, liver, kidney and heart of these mice (Supplementary Figure S8B). These results suggest that this combination does not exert generalized toxicity and effective in retarding tumor progression in mice.

## DISCUSSION

**Figure 8:**
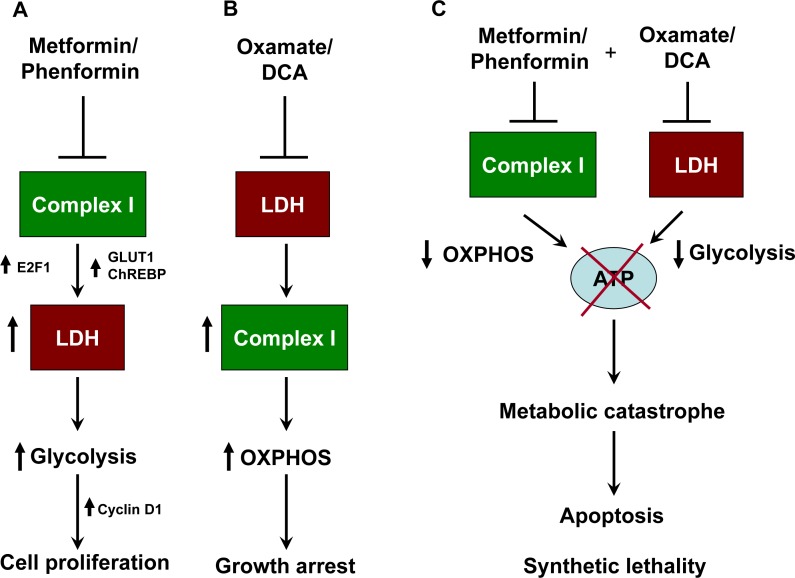
Schematic representation of synthetic lethality induced by combination of metformin and oxamate treatment in melanoma **A.** Inhibition of respiratory complex I by metformin or phenformin alone results in elevation of glycolysis owing to the activation of lactate dehydrogenase (LDH). Generation of building blocks and ATP exclusively through glycolysis helps in rapid tumor growth. **B.** Targeting LDH or lactate generation by oxamate and DCA or by siRNA causes activation of OXPHOS and subsequent ATP generation. However this leads to suppression of cell proliferation without inducing cell death. **C.** Blocking both the enzyme together induces metabolic catastrophe owing to the depletion of cellular ATP pool. This leads to the initiation of apoptosis, and suppression of tumor growth. Complex I and LDHA form a synthetically lethal pair in melanoma cells.

Cancer cells including melanoma cells divert huge amount of carbon flux to glycolysis [5-7]. This helps the cells to generate ATP and other building blocks for rapid tumor growth [5]. Several reports suggest that targeting metabolic pathways reduces tumor progression and cell growth *in vitro* [40-42]. In the present study, we demonstrate that metformin promotes melanoma growth by elevating glycolysis owing to the inhibition of complex I function, while inhibition of LDH causes growth arrest in cells. Inhibiting lactate generation in melanoma cells treated with metformin affects cell survival thereby causing reduction in tumor progression (Figure 8).

Consequences of inhibiting complex I on growth of cancer cells are not clearly understood though, reports suggest that complex I inhibition leads to cellular transformation and enhances cell growth [14-18]. Conversely, inhibition of complex I has also been reported to retard tumor progression in mice [30]. These diverse outcomes are likely to be dependent on the availability of nutrients in the tumor microenvironment. It has been reported that when glucose is available in abundance, inhibition of complex I promote glycolysis and rapid proliferation of cells, while it induces cell death under metabolic stress conditions [43]. In this study, we observed that inhibition of complex I by metformin promotes melanoma tumor progression in mice.

While metformin has been shown to inhibit proliferation of many cancer cells and also restricts tumor progression of xenograft in mice [33, 34], it is known to exert growth inhibitory as well as growth promoting effects in melanoma [35-37]. Interestingly, we detected that B16F10 and A375 derived tumors grew rapidly in mice administered with metformin and growth rate varied depending on glycemic status of mice (Figure 2D and 2E). This observation concurs with recent report demonstrating that metformin facilitates tumor progression of BRAF mutant melanoma cells [35].

Disparity in the action of metformin on the growth of melanoma cells *in vitro* and *in vivo* is likely to be influenced by the availability of glucose and lactate concentration which might influence its action. Metformin inhibits cell growth and induces apoptosis under glucose limiting conditions [43], while we noticed that in presence of high glucose it causes growth arrest without affecting cell death. We reasoned for this discrepancy that cancer cells derive ATP *via* OXPHOS under metabolic stress, whereas when glucose is abundant ATP is derived mainly through glycolysis. Inhibiting complex I under high glucose condition further promotes glycolysis resulting in excess lactate generation, and occurrence of this phenomena has been reported earlier [44]. Collectively, these findings suggest that growth arrest induced by metformin in melanoma cells *in vitro* is a consequence of acidification of medium due to excess lactic acid accumulation because of rapid utilization of available glucose. Notably, metformin induced growth arrest can be prevented by replenishing medium frequently to maintain optimum growth conditions, as metformin treated cells required more glucose to proliferate due to enhanced aerobic glycolysis. This mimics *in vivo* condition wherein probably rapid, constant circulation of access lactic acid allows cancer cells to use more glucose which helps in rapid proliferation. Moreover, lactate can also be utilized by cancer cells in tumor [45, 46]. Additionally, it has been reported that metformin induces angiogenesis *via* elevating VEGF level [35, 47]. We report that inhibition of complex I increases lactate production and it has been previously suggested that the glycolytic switch enhances angiogenesis [48, 49]. Our results show a positive correlation between serum lactate and VEGF level in mice administered with metformin. Therefore, it is likely that metformin-enhanced tumor growth is facilitated by lactate induced angiogenesis that can be mediated by VEGF [48]. Furthermore, we observed increased level of E2F1, an important cell cycle regulatory molecule, in the tumors excised from mice administered with metformin. In addition to promoting cell proliferation, E2F1 also regulates many genes involved in glycolysis [50] which is essential for rapid growth of cancer cells. Therefore, it is likely that, E2F1 might play an important role in mediating metformin-induced rapid tumor progression probably *via* regulating aerobic glycolysis.

Glycolytic inhibitors such as 2-deoxy glucose, lonimide, 3-bromopyruvate, DCA, and drugs interfering with metabolic pathways have shown promising outcome in suppressing tumor growth [40-42, 51-53]. In addition, targeting lactate generation pathway is appealing especially in glycolytic cancers [54, 55]. Melanoma cells are metabolically glycolytic, thereby rely primarily on the high activity of LDH for ATP generation [6]. Melanoma cells overexpress LDH and high serum level often correlates with poor prognosis and patient survival [3, 8-10]. Our results indicate that disrupting the conversion of pyruvate to lactate profoundly affects proliferation of melanoma cells. This is in agreement with the reports that tumors of glycolytic cell types are more susceptible towards inhibition of LDH-A with FX11 [56].

LDH plays an important role in metabolic homeostasis and tumor maintenance [12, 57]. We used oxamate and DCA to inhibit lactate generation in melanoma cells. DCA is an orally deliverable small molecule that has been used for treatment of lactic acidosis, and restoration of OXPHOS functionality by DCA has been shown to induce cell death [52, 53]. Moreover, DCA has previously been shown to reduce lactate production and triggers apoptosis in melanoma cells [58, 59]. Oxamate, an analog of pyruvate, is also used to inhibit LDH. A recent study by Miskimins et al., suggests that targeting complex I and LDH together can be a promising strategy to halt growth of aggressive cancers [60]. Concurrence with this, we also noticed that preventing lactate generation and simultaneously inhibiting complex I by oxamate/DCA or by LDH specific siRNA and metformin respectively results in depletion of cellular ATP pool. Decline in cellular ATP pool evokes metabolic catastrophe which leads to apoptosis in melanoma cells. Metabolic catastrophe-induced cell death is considered as a promising strategy to halt cancer progression [61]. Moreover, our study signifies the distinct role of complex I and LDH in cell proliferation, and these two constitutes ATP generating route either through OXPHOS or substrate level phosphorylation respectively. Blocking either of the enzymes alone does not induce cell death as cells can switch to alternative pathway for ATP generation. Whereas, simultaneously inhibiting both of these two, induces apoptosis. This clearly indicates that cells depend on the function of these two enzymes which form synthetically lethal pair, which is a promising phenomenon that can be employed for selective targeting of melanoma cells [62, 63]. Given that E2F1 is involved in regulating apoptosis [64], it is likely that the apoptosis induced by the combination of metformin and oxamate/DCA might involve E2F1. Metformin promotes apoptosis in cancer cells *via* activating p53 [37]. However, in case of melanoma, role of p53 is not clear. As the literature suggests, p53 is non-functional in melanoma and its levels are paradoxically elevated in advanced grades of melanoma [65-67]. Previously, we have reported that elevated level of p53 is associated with increase in tumor growth [68]. Therefore, it is unlikely that p53 is involved in apoptosis induced by the combination treatment and, thus, more studies are needed to evaluate the functionality of E2F1 and p53 in melanoma.

While using combinations of different drugs which can synergistically promote cancer cell death, this may also enhance toxicity to normal cell. Therefore, it is crucial to access the impact of the combination treatment on normal cells. Importantly, our data suggest that this combination is effective in killing cancer cells both *in vitro* as well *in vivo*, and has least impact on the survival of normal cells. The differential sensitivity between melanoma and normal cells due to metformin and oxamate/DCA combination can be attributed to the fact that melanoma cells are highly glycolytic and overexpress molecules involved in lactate generation and secretion as compared to normal cells [3, 6-10]. Interestingly, in comparison to normal cells, cancer cells display higher uncoupled mitochondrial respiration [31]. Cancer cells upon treatment with metformin exhibit greater compensatory elevation in glycolysis than normal cells [31, 32], thus weighing the metabolic susceptibility of cancer cells. Therefore, inhibiting LDH or lactate generation/secretion suppresses cancer cell growth while normal cells are least affected as sufficient ATP can still be produced from OXPHOS because metformin is a weak complex I inhibitor. As OXPHOS activity is higher in normal cells as compared to the melanoma cells, metformin and oxamate/DCA combination is likely to have least adverse impact on respiration of normal cells.

Our study opens new avenues in targeting metabolism of cancer cells and can be further implicated in testing other clinically approved drugs known to inhibit glycolysis along with metformin. The present study suggests that any drug/inhibitor that blocks lactate generation can be used in combination with metformin for the better management and preventing tumor growth. For example, rapamycin, a clinically approved drug is shown to prevent lactate generation [69, 70]. Similarly, it has been shown that inhibition of BRAF results in suppression of glycolysis [6, 7]. Therefore, rapamycin as well as BRAF inhibitors can be used along with metformin to improve therapeutic outcome with reduced side effects. Collectively, our results indicate that LDH or other mechanism which control lactate generation or secretion is critical for rapid melanoma progression under OXPHOS-compromised conditions, and this can be exploited as a suitable therapeutic target for controlling growth of glycolytic cancer cells. More extensive studies are needed to evaluate functionality of LDH and complex I in other cancer models, and subsequently their implication in cancer therapy in general.

## MATERIALS AND METHODS

### Animal experiments

Mice were procured from Experimental Animal Facility (EAF) at National Centre for Cell Science (NCCS), Pune, India. Hyperglycemia in mice was induced using STZ as previously described [71] with slight modifications. Briefly, male C57BL/6J and NOD/SCID mice were fasted for 6 h prior to intraperitoneal injection of STZ (50 mg/kg) in 0.01 M citrate buffer (pH 4.4) for three consecutive days. Blood glucose measurement was performed by nipping the tail and applying blood on to a glucose analyzer (Accu-Chek Active, Roche, Germany). Mice having blood glucose value over 200 mg/dl were considered as hyperglycemic. Tumor was induced by injecting B16F10 (2 × 10^5^) or A375 (1 × 10^6^) cells in 100 μl sterile PBS subcutaneously at right flank of C57BL/6J and NOD/SCID mice respectively. Tumor progression was monitored regularly by measuring its size with a digital caliper (Sigma, USA) following the appearance of palpable tumors. Oral administration of metformin on alternate days was initiated a week prior to tumor challenge in hyperglycemic mice. Otherwise metformin was given only after tumor reached to an optimum size and the treatment was continued till the completion of the experiment. At the end of the experiment, mice were sacrificed; tumors were excised and stored in either −80^°^C or in 10% formalin solution for further studies. In another set of experiment, mice (C57BL/6J background, male) were randomly divided into 4 groups following the appearance of palpable tumor. To examine the outcome of metformin treatment in combination with LDH inhibitor oxamate, mice were given orally either metformin (200 mg/kg) or oxamate alone (500 mg/kg) or both together on every alternate day. Tumor progression was regularly monitored with a caliper. At the end of experiment, tumor, muscle and liver tissues were excised, weighed and stored in −80^°^C for further analysis. All animal experiments have been performed as per the guidelines of the committee for the purpose of control and supervision of experiments on animals (CPCSEA), Government of India, and after obtaining permission of the Institute's Animal Ethics Committee (IAEC).

### Cell lines and culture conditions

B16F10 murine melanoma, A375 and SKMel28 human melanoma cells were purchased from ATCC (VA, USA) and maintained in our in house repository. Non-cancerous cells, AML12 (mouse hepatocytes), L6 (rat muscle cells) and MEF (mouse embryonic fibroblasts) were used in parallel along with cancer cells as a control. All the cells were grown in their respective medium containing either 1 mM or 25 mM glucose depending upon the experiments and supplemented with 10% heat inactivated fetal bovine serum (Hyclone, UT, USA), penicillin (100 U/ml) and streptomycin 100 μg/ml (Life Technologies, NY, USA), at 37^°^C in presence of 5% CO_2_.

### Chemicals and reagents

Metformin, phenformin, oligomycin, rotenone, decylubiquinone, streptozotocin (STZ), [3-(4, 5-dimethylthiazol-2-yl)-2, 5diphenylterazolium bromide] (MTT), NAD+, NADH, ATP, AMP, D-glucose, sodium oxamate, iodoacetate, 2-deoxy-D-glucose, dichloroacetate (DCA), Diaminobenzidine (DAB) and phloretin were purchased from Sigma (MO, USA). Antibodies for ChREBP (1:1000), GLUT1 (1:1000), LDHA (1:1000), Cyclin D1 (1:1000), PCNA (1:1000), CDK4 (1:1000), p21 (1:1000), E2F1 (1:1000) CD31 (1:100), PARP-1 (1:1000), Bcl-2 (1:1000), Bax (1:1000), β-tubulin (1:1000), GAPDH (1:1000), HSP60 (1:1000), and VEGF (1:50) were from Santa Cruz Biotechnology (CA, USA).

### Cell lysate preparation and immunoblotting

Cells were washed thrice with phosphate buffered saline (PBS) and lysed in lysis buffer containing 50 mM Tris-Cl (pH 7.5), 120 mM NaCl, 10 mM sodium fluoride, 10 mM sodium pyrophosphate, 2 mM EDTA, 1 mM sodium orthovanadate, 1 mM phenylmethylsulfonyl fluoride, 1% NP-40 and protease inhibitor cocktail (Roche, Germany). Tumor Lysates were prepared by chopping tumor tissues into fine pieces, washed five times with 0.85% saline solution containing protease inhibitor cocktail and lysed in lysis buffer by homogenizing with a tissue homogenizer (Sigma, USA) followed by sonication. Lysates were clarified by centrifugation at 15,000 rpm for 30 minutes. Cell lysates were prepared under chilled conditions. Approximately, 50-100 μg of protein from whole cell lysates were resolved on 8-12% SDS-polyacrylamide gel which was subsequently transferred onto PVDF membrane (Millipore, Germany). The membrane was probed with desired primary antibodies followed by HRP-conjugated secondary antibodies. Immunoblots were detected by luminal reagent (Santa Cruz Biotechnology). Whenever required, the blots were stripped by incubating the membrane at 50^°^C for 15 min in stripping buffer (62.5 mM Tris-Cl, pH 6.8, 100 mM mercaptoethanol and 2% SDS) with intermittent shaking. Membranes were washed thoroughly with Tris buffered saline (TBS), and were reprobed with desired antibodies.

### Immunofluorescence or confocal microscopy

Cells were plated in Labtek chambered slides (Nunc, USA) and allowed to grow for 24 h. Treatment of metformin and other inhibitors were given for desired time periods and concentrations. Cells were washed with chilled sterile PBS and fixed with 3.7% paraformaldehyde solution at room temperature for 10 min. These were then permeabilized with 0.025% Triton X-100 in PBS for 10 min and subsequently blocked with 5% BSA in PBS for 1 h at 37^°^C. Cells were incubated with 1:100 dilutions of primary antibodies in the blocking solution for 2 h at room temperature and washed with TBST (TBS containing 0.05% Tween-20) at least five times before being incubated with appropriate labeled secondary antibodies (1:200) in blocking solution for further 1 h at room temperature. After five washes with TBST, samples were layered with mounting medium containing DAPI (Santa Cruz Biotechnology, USA). Slides were sealed, examined under a confocal laser scanning microscope (LSM510 Carl Zeiss, Germany) and images were captured. Images were subsequently processed by LSM image analysis software.

### Immunohistochemistry and histopathology

Immunohistochemical and histopathology was performed according to the Malvi et al. [72]. Briefly, fine sections of tumor, and other organs were made with microtome fixed on glass slides and parafinized. For immunohistochemistry, slides were deparafinized in xylene solution twice for 10 min, and subsequently washed thrice with 100%, 95%, 70% and 50% ethanol. Slides were again washed with distilled water followed by washing with PBS for 5 min. For antigen retrieval, slides were boiled in sodium citrate buffer (0.01 M, pH 4.5) in microwave oven for 10 min and allowed to cool at room temperature for further 20 min. BSA or normal goat serum (2%) was used for blocking in humidified chamber or in cold box for 1 h. Slides were probed with desired antibodies (1:100 dilution) in PBST (PBS containing 0.025% Tween-20) containing 0.01% BSA for 2 h at room temperature or overnight at 4^°^C. Slides were washed with PBST 4 times for 5 min, and were probed with compatible HRP-conjugated secondary antibodies for 1 h. These were again washed and stained with Diaminobenzidine (DAB) for 10 min followed by washing and counterstaining with hematoxylin. Slides were further washed with water, dehydrated with absolute alcohol followed by layering with mounting medium, and then analyzed for the expression of desired molecules. For histopathology, deparafinized slides were stained with hematoxylin and eosin, and microscopic analysis for cell density, cellular morphology, metastasis, cytotoxicity and necrosis was performed by pathologists in a blinded manner at KEM Hospital Pune, India.

### Cellular cytotoxicity and survival assay

Approximately 5 × 10^3^ (B16F10) and 10 × 10^3^ (A375 and SKMel28) cells were seeded in each well of 96 well tissue culture plates and allowed to adhere for 24 h at 37^°^C. Cells were treated as per the experimental requirement and proliferation or viability was assessed by MTT assay. MTT (50 μl, 1 mg/ml in DMEM without phenol red) was added to each well and incubated for 4 h at 37^°^C. Formazan crystals were solubilized in 100 μl of isopropanol by incubating with shaking at room temperature for 5 min. Absorbance was taken at 570 nm using 630 nm as reference filter. Untreated cells were considered as control (100% cell survival).

### Detection of apoptosis by annexin V staining

Cells were seeded at a density of approximately 3 × 10^5^ cells in 35 mm plates and allowed to grow for 24 h. Cells were treated with or without oxamate and DCA either alone or with metformin for 48 h or as the experimental requirement. Cells were harvested by trypsinization and processed for flow cytometry. Apoptosis was detected by dual staining of Annexin V and PI using apoptosis assay kit (BD Bioscience, USA) according to manufacturer's instructions.

### Cell cycle analysis

Cells were seeded at a density of approximately 3 × 10^5^ cells in 35 mm plates and allowed to grow for 24 h. Cells were treated with or without metformin, oxamate and other glycolytic inhibitors alone or together as indicated for 48 h or as experimental requirement. Cells were harvested by trypsinization and processed for flow cytometry. Briefly, cells were washed with chilled PBS and fixed in 70% ethanol on ice for 30 min. Following RNase (200 μg/ml) treatment for 30 min at 37°C, 50 μg/ml PI was added to cell pellet and incubated in dark for 30 min on ice. The fluorescence of PI was recorded through a 585 nm filter in a flow cytometer (FACS Calibur, Becton Dickinson, California, USA). Data were analyzed using Cell Quest Pro software for 10,000 cells.

### Long term cell survival assay

Cells (5 × 10^2^) were plated in 12 well plates for 24 h. Cells were treated without or with 25 mM or 50 mM oxamate and 10 or 20 mM DCA along with metformin and continued for further 48 h. Subsequently, medium was replaced with fresh drug-free medium. Plates were incubated for an additional 10-15 days at 37°C in CO_2_ incubator with medium change on every 2-3 days. Cells were then fixed (3% paraformaldehyde and 0.02% glutaraldehyde in PBS) and stained with 0.05% crystal violet.

### Glucose utilization assay

Cells (3 × 10^5^) were cultured in DMEM containing 25 mM glucose. After 24 h, medium was replaced with respective medium containing increasing concentration of metformin (0.1 mM, 0.5 mM, 1 mM, and 2 mM) or rotenone for 24 h and residual glucose present in the spent medium was monitored using GOD-POD based glucose assay kit (Spinreact, Spain) according to manufacturer's instructions. For measuring glucose utilization in the presence glycolytic inhibitors, cells were treated with 50 mM oxamate, 100 μM phloretin, and 20 mM DCA, either alone or together with 2 mM metformin or 100 μM phenformin. Consumed glucose was estimated by subtracting the remaining glucose in the medium from the initial concentration in control medium (450 mg/dl). The experiments were performed at least in triplicate and values were normalized to total number of cells.

### Lactate estimation assay

Lactate concentration in spent medium collected from the cells treated with or without metformin, oxamate and other glycolytic inhibitors, was determined using commercially available lactate estimation kit (Spinreact, Spain) as per the manufacturer's instructions. Briefly, culture medium or serum was diluted in 0.9% saline up to 10 times and 10 μl of sample was added to each well of 96 well ELISA plate. 150 μl of reagent provided with kit was added to each sample containing wells keeping unspent medium and reagent alone as blank and absorbance was recorded at 405 nm using a spectrophotometer (Thermo-Scientific, USA). Experiments were done at least in triplicate and the final values were normalized with total number of cells.

### siRNA transfection

Specific siRNA against LDHA was purchased from Santa Cruz Biotechnology (USA). Transfection was done using Lipofectamine 2000 (Life Technologies, USA) according to manufacturer's instructions. In brief, cells were plated at approximately 60% confluency. Next day, medium was replaced with OptiMEM (Life Technologies, USA) and kept for 3 h. Desired siRNAs were dissolved in buffers provided along with them. Lipofectamine2000 and siRNA were diluted separately in OptiMEM and incubated for 5 min. Thereafter, diluted reagents were mixed and further incubated for 30 min at room temperature. The resultant precipitate was left on the cells for 6 h, after which fresh DMEM supplemented with 10% FBS was added and incubated for further 24-36 h. Transfection efficiency was assessed by simultaneously transfecting pEGFPN1 plasmid. Immunoblotting was performed to ensure inhibition of respective gene expression.

### Preparation of mitochondria rich fraction from cells and tissues

Mitochondria rich fraction was prepared from cultured cells and tumors or normal tissues as previously reported [73]. Briefly, cells (1 × 10^6^) were plated in 10 cm plate and were trypsinized following treatment with the desired inhibitors for 24 h. Cell suspension was subjected to three cycles of freeze-thawing in hypotonic buffer (20 mM potassium phosphate). This suspension was centrifuged at 50,000 rpm for 1 h to obtain supernatant rich in mitochondria. Similarly, to isolate mitochondria from tissues, frozen tissues were chopped into fine pieces and homogenized in homogenization buffer (0.5 M Tris buffer pH 7.5, 100 mM EGTA, and 250 mM sucrose), followed by cyclic freeze-thaw procedure and centrifuged at 50,000 rpm for 1 h. Supernatant was collected as mitochondrial fraction and used for the determination of mitochondrial function and OXPHOS enzyme activity.

### Enzyme assays

Cells were homogenized in hypotonic (20 mM) potassium phosphate buffer (pH 7.5) containing protease inhibitor cocktail (Roche, Germany), vortexed and lysed by three cycles of freeze and thaw procedure. For preparing extract from tissues, tumor samples were chopped into fine pieces and homogenized in homogenization buffer (0.1M Tris, 0.1 M KCl, 350 mM EDTA and 1 M sucrose, pH 7.5) using tissue homogenizer. Homogenates were clarified by centrifuging at 10,000 rpm for 10 min at 4^°^C and kept on ice until the assays are performed.

*LDH activity assay:* Lactate dehydrogenase activity in cell lysates or tumor extract and serum was determined using LDH activity assay kit (Spinreact, Spain) according to manufactures instructions.

*Complex I activity assay:* Mitochondrial OXPHOS complex I enzyme assay was performed as described elsewhere [73]. Briefly, mitochondrial rich fraction of cell or tissue homogenate (20-50 μg of protein from tissue homogenate or 10-20 μg of mitochondrial rich fraction) was added to 700 μl of distilled water taken in a 1 ml cuvette, followed by addition of 100 μl potassium phosphate buffer (0.5 M, pH 7.5), 60 μl of fatty acid-free BSA (50 mg/ml), 30 μl of KCN (10 mM) and 10 μL of NADH (10 mM). Final volume was adjusted to 994 μl using distilled water, mixed by inverting the cuvettes and the baseline reading was acquired at 340 nm for 2 min. Reaction was started by adding 6 μl of decylubiquinone (10 mM, Sigma, USA) mixed properly by inverting the cuvettes. Decrease in the absorbance at 340 nm was followed for 10 min. Similar procedure was followed for the calculation of complex I activity in presence 2 mM metformin and 100 μM phenformin. Rotenone (10 μM) was used as a positive control. Final values were normalized with total cellular protein content.

### Total cellular ATP measurement

ATP level was measured by using commercially available ATP bioluminescence kit (Roche, Germany) according to manufacturer's instructions. Briefly, cells were grown in the presence of indicated drugs. Cells were harvested and lysed in 100 mM Tris-EDTA buffer containing 0.01% NP-40 and boiled for 1 min followed by thee freeze-thaw cycle. Luminescence was recorded for blank as well as for samples. For measuring ATP generated exclusively through OXPHOS and glycolysis, cells were treated with oligomycin (10 μM) and oxamate or DCA respectively with or without metformin. Experiments were done in triplicate and repeated at least once, and final values were normalized with total protein contents.

### Statistics

Statistical analysis was performed using Sigma Plot 12.0 (Systat Software Inc., CA, USA). Most of the experiments were repeated at least once with minimum in triplicate unless otherwise mentioned. Data was represented as mean ± SD except for the indicated experiments. Wherever required, either paired or unpaired, two tailed student *t*-test was employed in the experiments assuming unequal variance unless otherwise mentioned, to calculate p-value. The values *p < 0.05, **p < 0.01, ***p < 0.001 denote significant differences between the groups (*n* > 3 at the least).

## SUPPLEMENTARY MATERIAL FIGURES


