# Supplementary material for: Targeting metabolic flexibility by simultaneously inhibiting respiratory complex I and lactate generation retards melanoma progression
Source: Oncotarget. 2015 Oct 15;6(35):37281–99. doi: 10.18632/oncotarget.6134 (PMC4741930; doi:10.18632/oncotarget.6134)
Supplement: Supplementary file 1 [file oncotarget-06-37281-s001.pdf]

Targeting metabolic flexibility by simultaneously inhibiting respiratory complex I and lactate generation retards melanoma progression

Supplementary Material

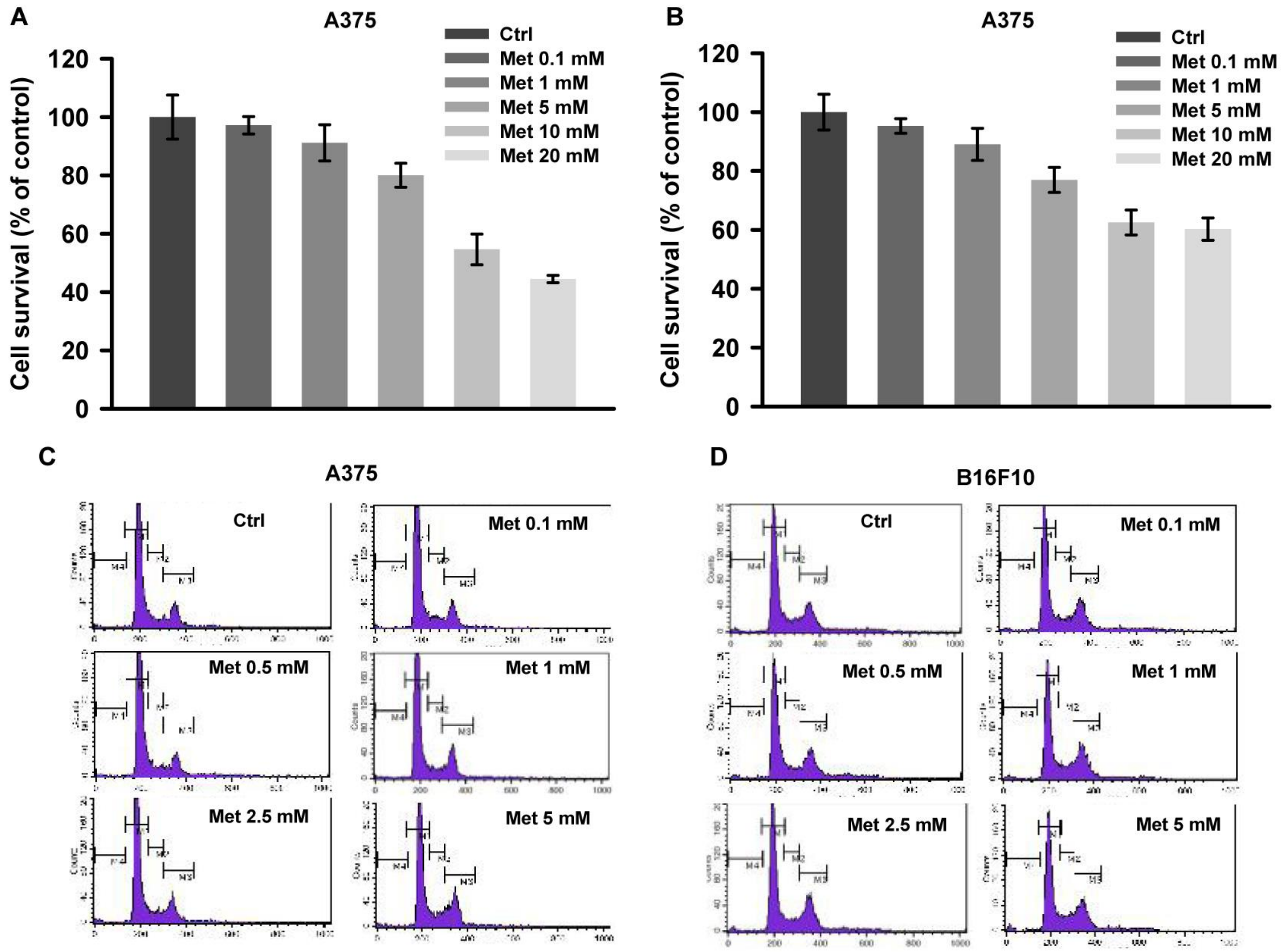

Supplementary Figure S1

Supplementary Figure S1: Metformin induces growth arrest in melanoma cells *in vitro*. (**A** and **B**) A375 and B16F10 cells were treated with indicated concentration of metformin for 48 h and inhibition of cell proliferation was measured by MTT assay. (**C** and **D**) A375 and B16F10 cells were treated with indicated concentration of metformin for 48 h and cell cycle was determined by PI staining through flow cytometry. (Ctrl- control, Met- metformin)

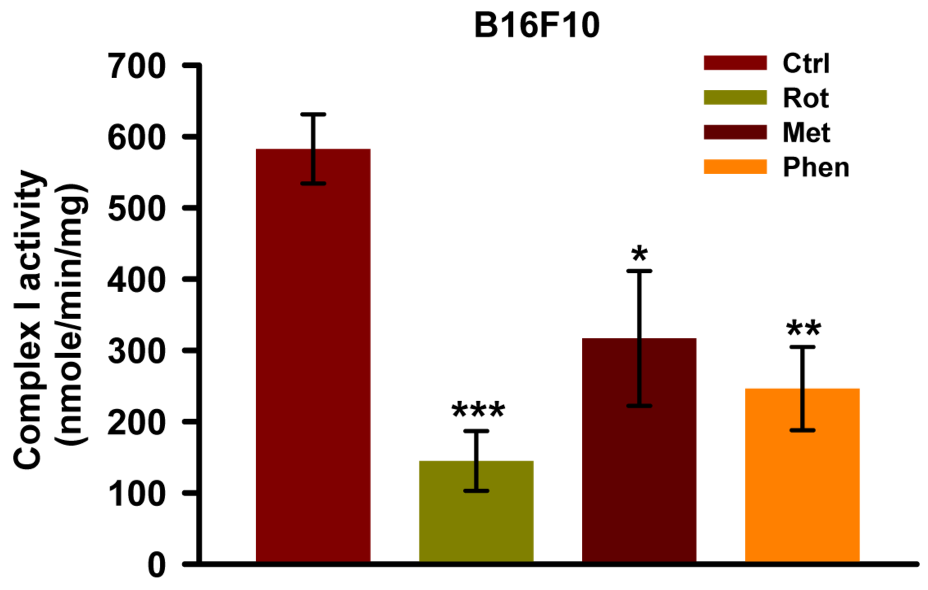

Supplementary Figure S2

Supplementary Figure S2: Metformin and phenformin inhibits complex I activity *in vitro.* Mitochondrial rich fraction of B16F10 cells was incubated with 2 mM metformin and 100 μM phenformin separately and complex I activity was determined as mentioned in methods section. Rotenone (10 μM) was used as a positive control. Data was represented as the mean ± SD. The values $*p < 0.05$, $**p < 0.01$, $***p < 0.001$ denote significant differences between the groups. (Ctrl- control, Rot- rotenone, Met- metformin, Phen- phenformin)

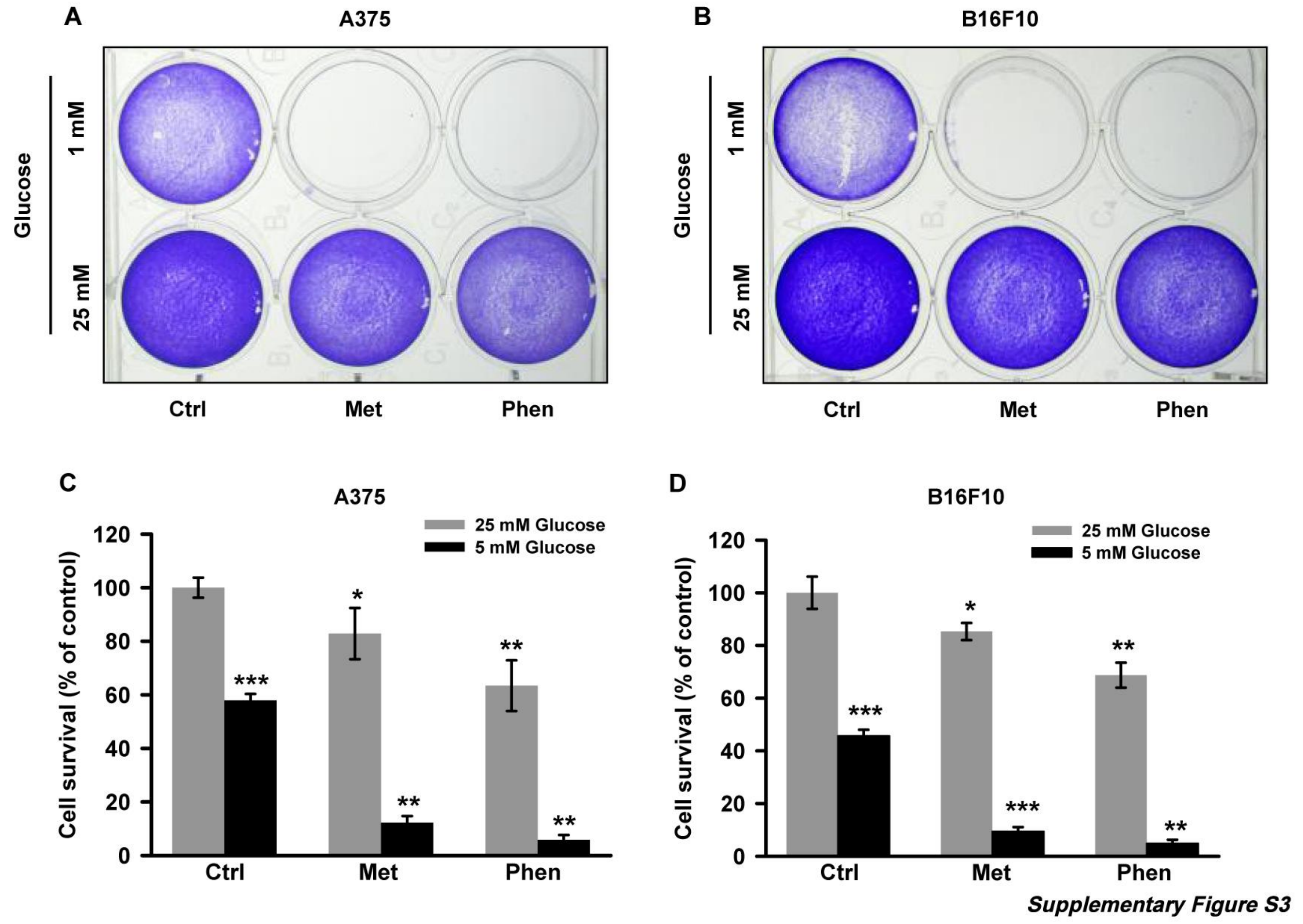

Supplementary Figure S3: Glucose influences impact of metformin on the growth of melanoma cells. (**E** and **F**) Representative image showing the survival of A375 (**E**) and B16F10 cells (**E**) grown in DMEM containing either 1 mM or 25 mM glucose in presence or absence of metformin and phenformin. Cells were treated with 2 mM metformin and 100 μM phenformin for 48 h. Cells were stained with crystal violet and photographed. (**G** and **H**) Cell survival in A375 and B16F10 cell grown under similar conditions mentioned in (E and F). Inhibition in cell proliferation was measured by MTT assay. All values are represented as mean ± SD. The values $*p < 0.05$, $**p < 0.01$, denote significant differences between the groups. (Ctrl- control, Met- metformin, Phen- phenformin)

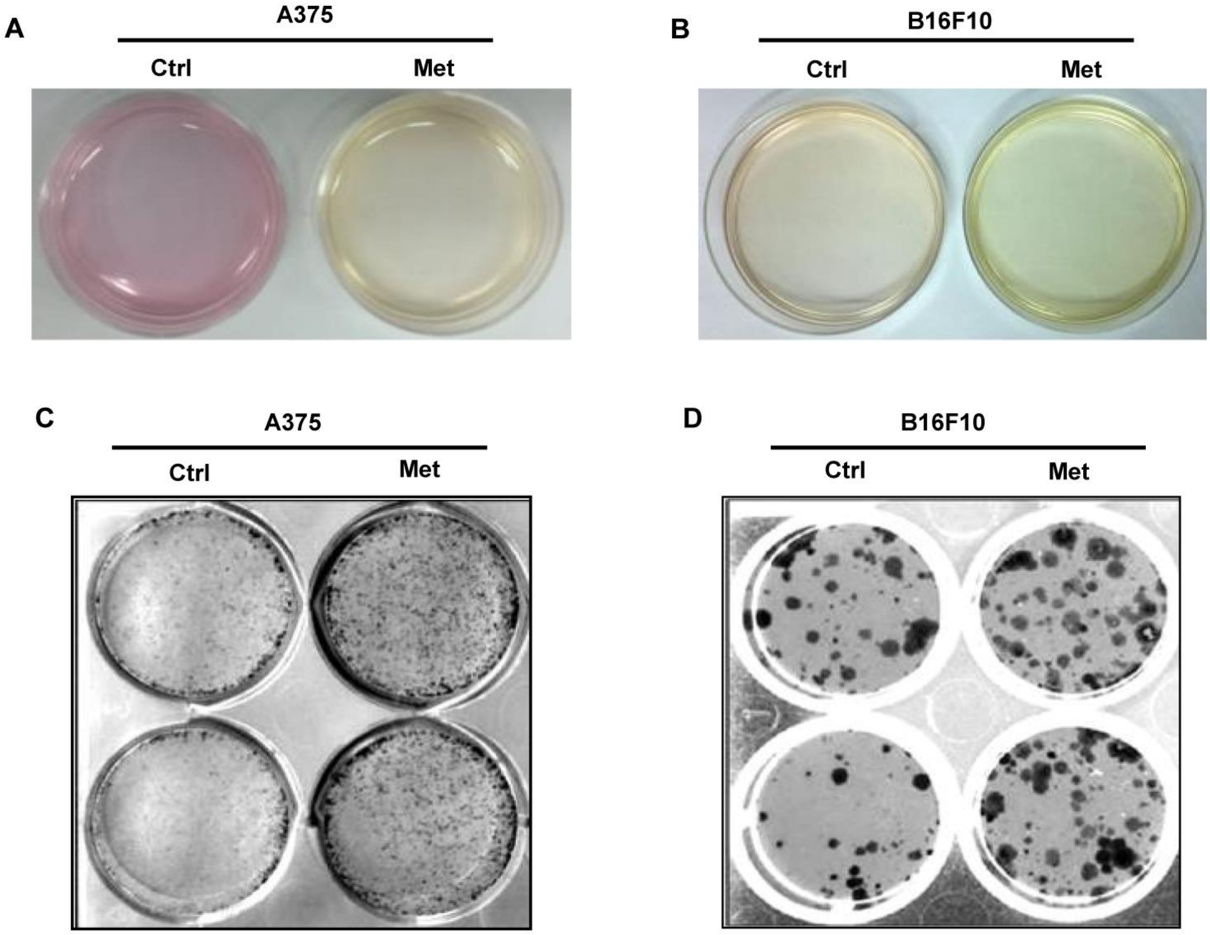

Supplementary Figure S4

Supplementary Figure S4: Metformin induces growth arrest in vitro owing to the extracellular acidification. (**A** and **B**) Equal number of A375 and B16F10 cells was grown in 35 mm culture dish in presence or absence of 2 mM metformin for 48 h. Representative photograph shows change in medium colour due to decreased extracellular pH. (**C** and **D**) Long term survival of A375 and B16F10 cells treated with metformin. Cells were growth in presence of metformin for 24 h and medium was replenished with fresh medium and further grown for 10 days. (Ctrl- control, Met-metformin)

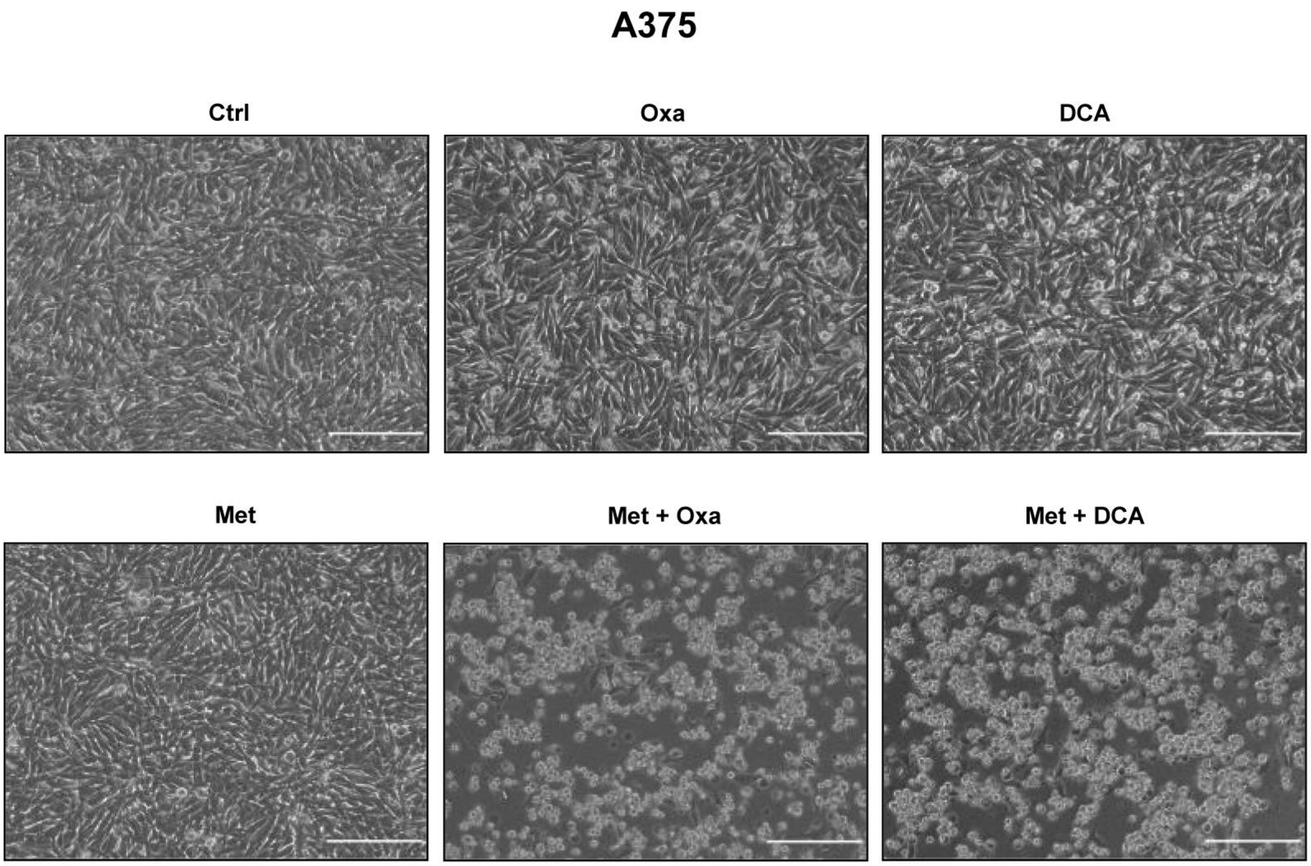

Supplementary Figure S5

Supplementary Figure S5: Inhibition of complex I and lactate generation together causes cell death in melanoma cells. A375 cells were grown in DMEM containing 25 mM glucose. Cells were treated with 50 mM oxamate or 20 mM DCA either alone or with 2 mM metformin for 48 h. Representative image showing change in cellular morphology of A375 cells treated with oxamate or DCA together with metformin. (Ctrl- control, Met- metformin, Oxa- oxamate)

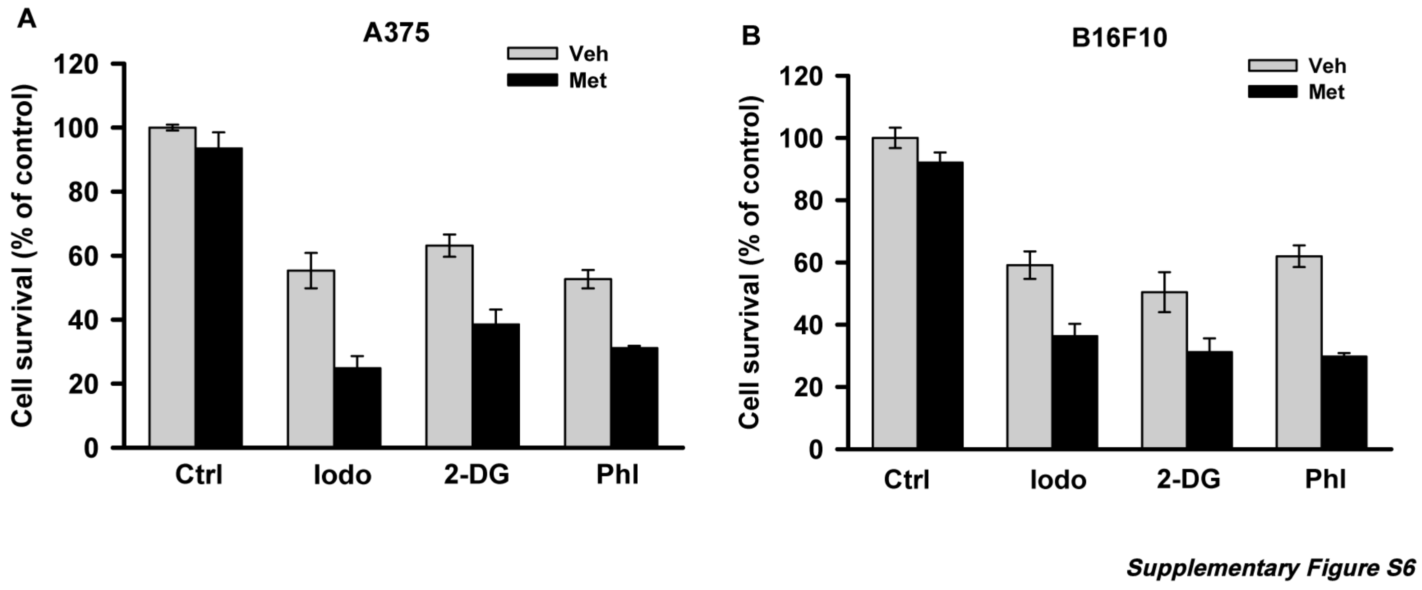

Supplementary Figure S6: Blocking glycolysis and complex I results in growth suppression in melanoma cells. (**A** and **B**) A375 and B16F10 cells were treated with 1 mM iodoacetate, 10 mM 2-DG and 100 μM phloretin either alone or together with 2 mM metformin for 48 h. Cell survival was accessed by MTT assay. (Ctrl- control, Iodo- iodoacetate, 2-DG- 2 deoxy D-glucose, Phl- phloretin)

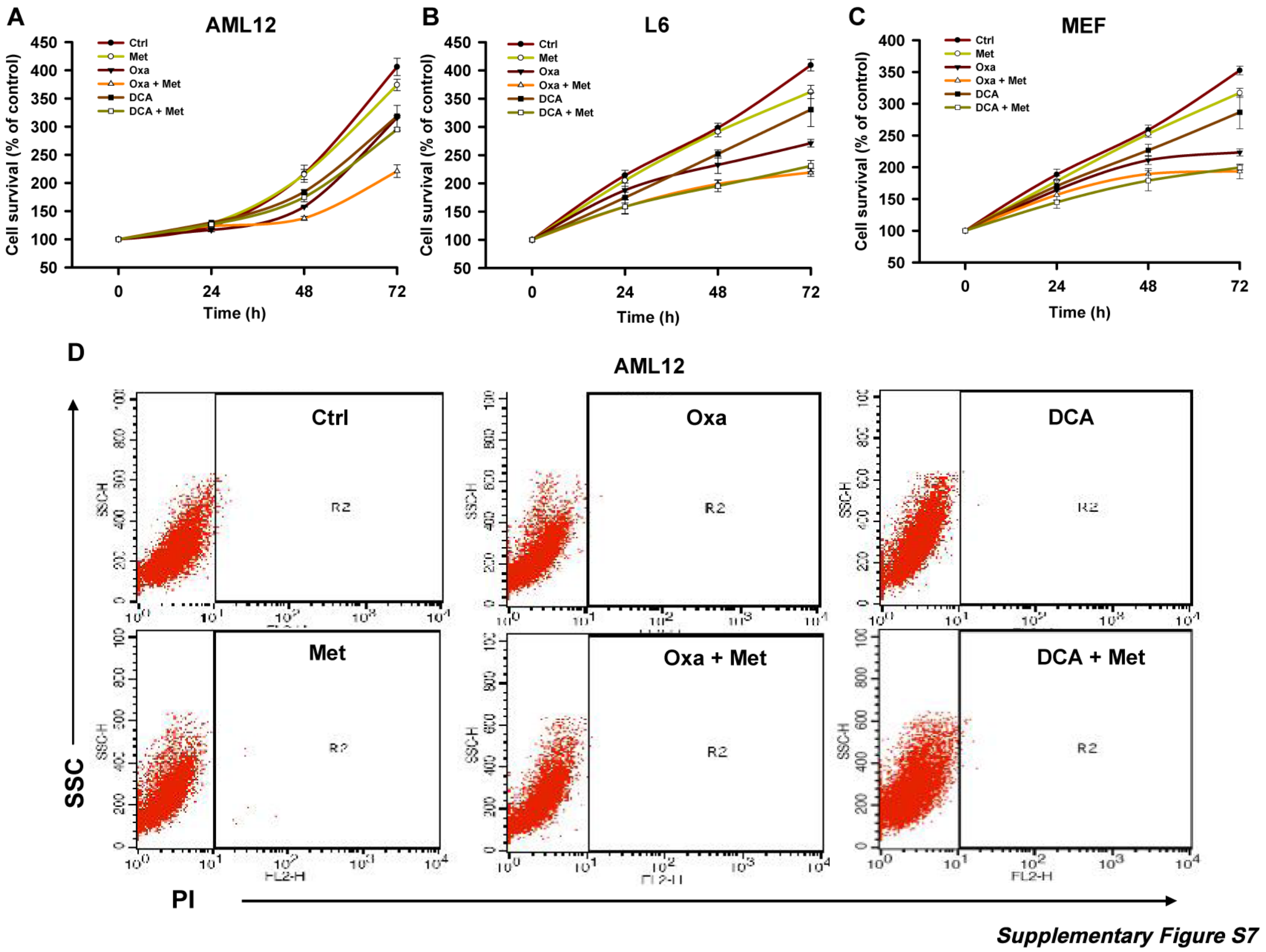

Supplementary Figure S7: Combination of oxamate and metformin does not influence survival of non-cancerous cells (**A**-**C**) AML12 (mouse hepatocyte), L6 (muscle cells) and MEFs (mouse embryonic fibroblast) cells were treated 25 mM oxamate and 10 mM DCA either alone or together with 1 mM metformin for indicated time. Cell survival was accessed by MTT assay. (**D**) AML12 cells were grown in presence or absence of oxamate or DCA either alone or in combination with metformin for 24 h. Cell death was accessed by propidium iodide via flow cytometry. (Ctrl- control, Met- metformin, Oxa- oxamate)

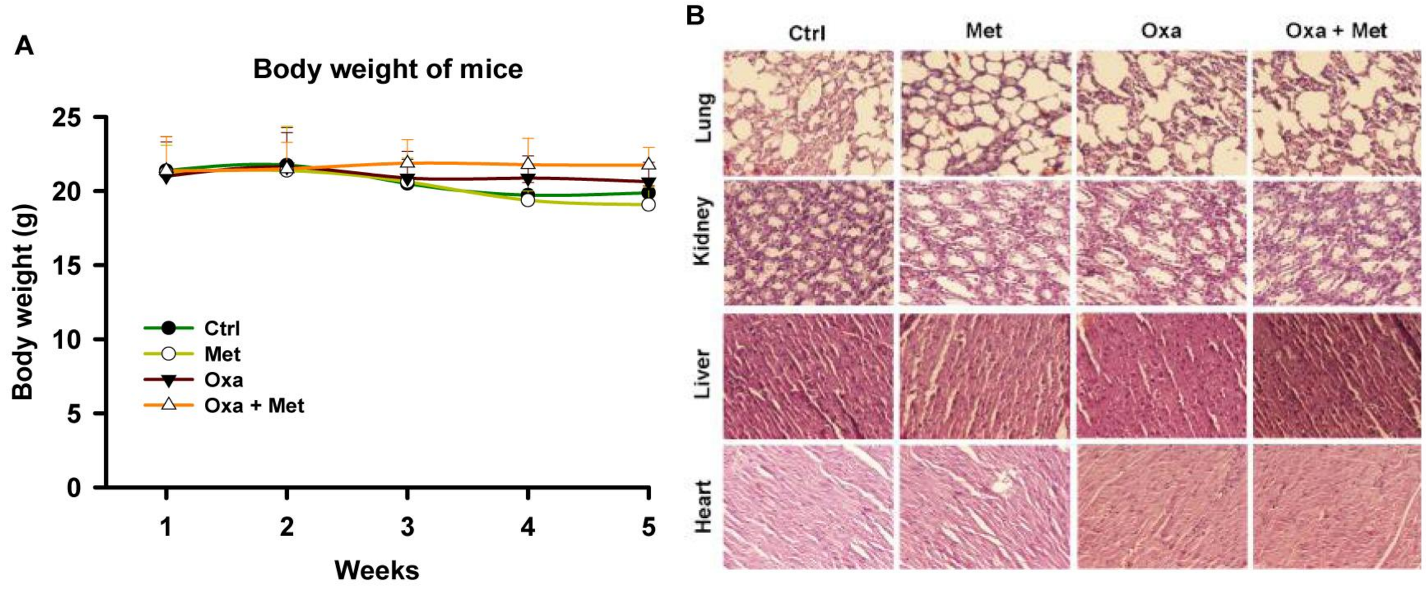

Supplementary Figure S8

Supplementary Figure S8: Combination of oxamate and metformin does not have generalized toxicity in mice. (A) Body weight of mice administered with indicated either metformin or oxamate alone or together in combination. **(B)** H&E staining of tissue section representing histology of vital organs of mice administered with indicated treatment. Organs (lungs, kidney, liver and heart) were excised from the mice with indicated treatment groups and immediately kept in 10% formalin solution for processing of histopathology slides. Tissue sections were stained with hematoxylin and eosin (H&E) image were taken using microscope equipped with DP71 camera (Image magnification= 100 X).
